# The Healing Effect of Scrophularia Striata on Experimental Burn Wounds Infected to Pseudomonas Aeruginosa in Rat

**Published:** 2015-01

**Authors:** Nader Tanideh, Mohammad Hossein Haddadi, Mohammad Hossein Rokni-Hosseini, Masood Hossienzadeh, Davood Mehrabani, Kourosh Sayehmiri, Omid Koohi-Hossienabadi

**Affiliations:** 1 Shiraz Burn Research Center, Pharmacology Department, Shiraz University of Medical Sciences, Shiraz, Iran;; 2 Student Research Committee, School of Medicine, Shiraz University of Medical Sciences, Shiraz, Iran;; 3 Pathology Department, Shiraz University of Medical Sciences, Shiraz, Iran;; 4 Stem Cell and Transgenic Technology Research Center, Shiraz University of Medical Sciences, Shiraz, Iran;; 5 Prevention of Psychosocial Injuries Research Center, Ilam University of Medical Sciences, Ilam, Iran;; 6 Laboratory Animal Center, Shiraz University of Medical Sciences, Shiraz, Iran

**Keywords:** *Scrophularia striata*, Wound, Healing, Silver sulfadiazine, *Pseudomonas aeruginosa*

## Abstract

**BACKGROUND:**

The cause of death in burn patients after 48 hours of hospitalization has been reported to be bacterial infections. Recently, due to the compounds accelerating the healing process and the intense reduction of treatment side effects, medicinal plants are used to cure burn wound infections. This study aims to investigate the medicinal effect of the ethanolic extract of *Scrophularia striata *on burn wound infection in *in-vivo* and *in-vitro* in comparison with silver sulfadiazine (SSD).

**METHODS:**

One hundred and fifty male Sprague Dawley rats were divided into 3 equal groups. A hot plate of 1×1cm was used to create second degree burn wounds. The ethanolic extract of *S. striata* was provided through percolation method. Group 1 was treated with SSD, group 2 with *S. striata*, and group 3 was considered as control group. All animals were infected to *Pseudomonas aeruginosa*. On days 3, 7, 10, 14, and 21 after burn wound injury, the animals were euthanized and were evaluated histologically. The MIC and MBC were determined using the micro dilution method.

**RESULTS:**

The rate of wound healing was significantly greater in *S. striata* group in comparison to SSD and control groups.

**CONCLUSION:**

*S. striata* contains was shown to have anti-bacterial and wound healing effects while this effect was significantly more than SSD denoting to its use when needed for burn wounds infected to *P. aeruginosa*.

## INTRODUCTION

Burn wound infection is the major cause of disability and mortality affecting all ages groups in both developed and developing countries.^1^^,^^2^ Death in the first 48 hours of hospitalization is caused by organ failures and burn shocks which are dependent upon the type of burn and total body surface area (TBSA). However, the causes of death after 48 hours are nosocomial infections, wound infections, and septicemia.^3^ Disappearance of the primary defenses provides favorable conditions for implantation and invasion of opportunistic pathogens that can cause infection in a short time.^4^ The most common infectious bacterium that causes septicemia is *Pseudomonas aeruginosa*.^5^

Wound healing is widely discussed in the medical literature. To treat burn wound infections, a variety of common ointments, vaccines and antibiotics are used. Despite high side effects, topical medications like silver sulfadiazine (SSD) are most frequently used in healthcare centers.^6^^,^^7^ In topical burn therapy, SSD was introduced as the gold standard having antibacterial properties too.^8^ Using conventional antibiotics has led to an increase in multi-drug resistant strains of *Pseudomonas aeruginosa*, which is one of the most worrying opportunistic factors in nosocomial infections.^9^

Numerous studies were carried out to develop more sophisticated dressings to expedite healing process and diminish bacterial burden in wounds. Even medicinal plants were introduced in wound healing of burned injuries, traditional forms of medicine, especially herbal products, which have been employed for centuries in Africa and Asia are under scientific investigation for their roles in wound treatment.^10^^-^^14^

In western states of Iran, a plant of Snapdragon with the scientific name of *Scrophularia striata Boiss* has traditional medical usage. Different extracts of this plant are traditionally used in treating infectious diseases. Pharmaceutical activities ranging from reduced edema, reduced infiltration, and proliferation in connection with T-cells have been studied. The ingredients of this medicinal plant prevent the release of inflammatory factors such as PGE-2, IL-4 and IL-1B. These ingredients are used in treating inflammatory diseases.^15^^,^^16^ Probably, the anti-bacterial effects of this plant are related to its phenolic, flavonoid, and flavonol compounds, the highest amount of which having been isolated in the ethanolic extract. These components are attached to the bacterial outer membrane proteinsand deactivate the matrix metalloproteinase. The research conducted on this subject indicate that the anti-bacterial effect of this plant is considerably higher than that of conventional antibiotics.^17^^-^^19^

Regarding the deadly risks of *Pseudomonas* infections, long-lasting therapy period, and side effects of treatment with SSD, a therapy based on medicinal plants with multiple anti-bacterial and healing effects can be a suitable alternative.^11^^,^^14^ Regarding to curative properties that have been suggested, we decided to investigate the anti-bacterial and healing effect of the ethanolic extract of the plant *S. striata* on *P. aeruginosa* burn wound infection in comparison to SSD in experimental rats.

## MATERIALS AND METHODS

One hundred and fifty male Sprague Dawley rats (250±30 grams) from the Laboratory Animal Center of Shiraz University of Medical Sciences, Shiraz, Iran were enrolled. The whole stages of the investigation were completed based on the rules posed by the Animal Care Committee of Veterinary Organization of Iran and confirmed by this committee too. All the rats were kept in separate racks at 22-25˚C, 50% humidity and 12-hour cycle of light–darkness and these were fed ad libitum.

*S. striata *was collected from the natural habitat of the plant in west of Iran (Ilam Provine) with voucher number of 24998 from the Herbarium Department of the Medicinal Plants Research Center of Shiraz University. Plants were dried out and the ethanolic extract was provided through percolation method, condensed in vacuum by distillation.

The animals were divided into two treatment groups of SSD (Razi Pharmaceutical Company, Iran) and *S. striata* and one control group. First, the animal was anesthetized using ketamine (100 mg/kg) and xylazine (10 mg/kg). After removing the hair at the animal’s back with a shaver, the skin was washed with cotton and savlon for disinfection. In order to create second-degree burn wounds, a hot plate of 1×1cm was used. The created burn was confirmed by a pathologist based on TBSA.^20^ All the rats were kept in separate cages. The rats were all divided into 6 equal subgroups. In SSD group, half a gram of the 1% ointment; in *S. striata* group, 1 ml of the 10% *S. striata* dilution; and in the control groups, only topical base gel were administered on the wound surfaces. 

In this study, a standard strain (ATCC27853) and also a clinical strain of *P. aeruginosa* isolated from a patient with the infection caused by second-degree burn wounds (from Ghotb-Al-Din Burn Hospital, Shiraz, Iran) were used. From both standard and clinical strains, the fresh standard McFarland dilution 1 [10^8^ colony forming units (CFU)] was provided to be injected to the burn area. To infect the animals, each rat was injected with 0.1 ml of bacterial dilution in the wound area topically. After 24 hours, the wound area was sampled using a sterile swab to confirm *P. aeruginosa* infection. The bacterial strains and the infection caused by wound were confirmed by a bacteriologist throughout the study.^20^

To determine the wound healing, two methods of dimension determination and histological evaluation were used. In histological study, the animals were euthanized in time intervals of 14 and 21 days after therapy and tissue samples were taken from the edges of the infected wounds. The sample was then transferred to the laboratory in a 10% neutral-buffered formalin dilution for histological evaluation and investigated in terms of scoring criteria such as epithelialization, collagenization, granulation, inflammation, and neovascularization illustrated by Abramov et al.^21^

**Fig. 1:**
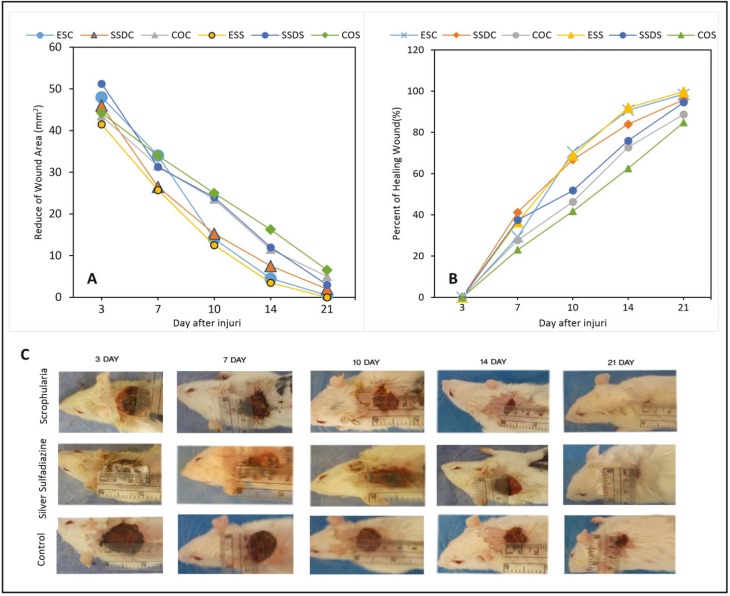
A: Comparison of wound healing according to mm^2 ^in different time intervals and various treatment groups of *Scrophularia striata, *silver sulfadiazine and the control; B: Comparison of the percentage of burn wound healing in different time intervals and various groups; C: The macroscopic wound healing process in treatment groups with respect to the time interval

To determine the dimensions of the wound from the day of burn (day 0) to the 21_st _day, each treatment-infection group in the interval subgroup of 21 day was measured with ruler based on mm in the whole time intervals of the wound (Figure 1). The data entered the EXCEL program and each wound dimension was determined using the *horizontal diameter×vertical diameter×*Л formula. Using the following formula, the wound healing percentage in each rat was determined:

$\mathrm{Wound percentage}=100\times \frac{\mathrm{wound surface on the mentioned day}}{\mathrm{surface on the first day}}$

Treatment percentage=100–wound percentage 

To determine the amount of Minimal Inhibitory Concentration (MIC) and Minimal Bactericidal Concentration (MBC), the modified micro dilution method suggested by NCCLS (National Committee for Clinical Laboratory Standards) was used.^22^ First, a suspension of both bacterial strains with the density equal to 0.5 McFarland was provided in Trypticase Soy Broth medium (Merck, Homburg, Germany). Then, as a result of continuous dilutions by *S. striata* and SSD in a density range of 20–200 mg/ml (20, 40, 60, 80, 100, 120, 140, 160, 180, 200), the two strains were heated at a temperature of 37˚C. After overnight incubation, the amount of MIC was determined due to the lowest concentration of anti-microbial agent that entirely inhibited the growth of the bacterium. The amount of MBC was determined by subculturing of the last clear MIC tube on to MacConkey agar and evaluation for bacterial growth. Finally, the results obtained were compared descriptively.^23^

Statistical analysis was done by SPSS siftware (Version 21, Chicago, IL, USA). Logistic-Regression, Chi-Square, Fisher’s exact and Kruskal-Wallis tests were used to analyze the data. While using the χ2 test was not possible, the Monte Carlo method was used to compare the *p* values. To compare neovascularization based on time and group, two-way ANOVA was used. Statistical values ​​*p*<0.05 were considered significant.

## RESULTS

**Fig. 2:**
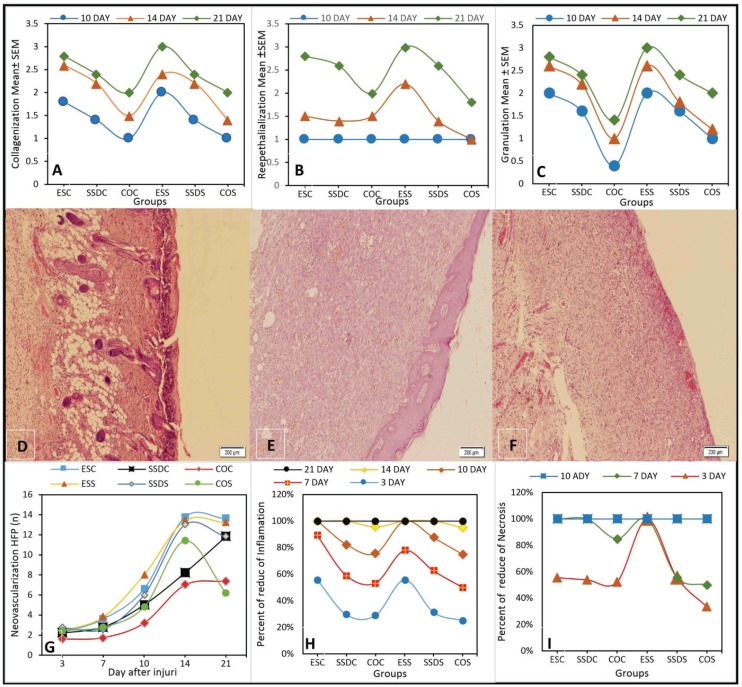
A-C: The values of remodeling criteria including collagenization, reepethelialization and granulation in different time intervals of 14, and 21 days and various treatment groups of *Scrophularia striata, *silver sulfadiazine and the control. D-F: Fourteen days after burn wound infection in various treatment groups of *Scrophularia striata, *silver sulfadiazine and the control, respectively (H&E×100); G: The increase of HPF (High Power Field) formation with respect to different time periods in various treatment groups of *Scrophularia striata, *silver sulfadiazine and the control; H and I: Comparison of inflammation and necrosis in various treatment groups of *Scrophularia striata, *silver sulfadiazine and the control at different time intervals.

The highest increment of regular and complete reepithelialization, particularly in the time periods of 14 and 21 days, was created in *S. striata* subgroups. This rate was 17% and 24%, respectively, that was higher than that of SSD subgroups (12% and 12.5%, respectively) (Figure 2). The formation of epithelium in the control on the 14_th _and 21_st_ days was either poor or partial (Figure 2). We found a significant association between the time periods and epithelialization (*p*<0.001).

The treatment groups and time periods under investigation had a significant relationship with collagenization (*p*<0.001). The highest collagenization was seen in *S. striata* subgroupsafter 14 and 21 days (28% and 30%, respectively). This rate was much higher than that of SSD subgroups after 14 and 21 days (12% and 13%, respectively) (Figure 2).

Immature granolation was seen in the control group on the 14_th _day. The highest amount of granolation in different time periods was seen in the *S. striata* subgroups. It was only the *S. striata* group in which moderate mature granolation was seen on the 14_th _day and full mature on the 21_st _day (Figure 2). We found a significant relationship for granulation among different groups (*p*<0.001).

Vessel formation in the wound area in the *S. striata* subgroups was more than others. This rate was completed in the two *S. striata* subgroups in a time period of 14 days. A significant relationship was seen between neovascularization and different time intervals and the subgroups (*p*=0.03). We found a significant association between the neovascularization changes and different time intervals and subgroups (*p*<0.001) (Figure 2).

In this study, the rate of necrosis and inflammation in the *S. striata* subgroups was very low, while it was seen in the SSD and control groups until the 14_th_ day. In the *S. striata* subgroups, no inflammation was seen after 14 days, while it was seen in the control group in the 21_st _day. Considering the statistical results, there was a significant relationship between inflammation and the groups in the investigation times (*p*<0.001) (Figure 2). The necrotic changes were significant in different times in various subgroups (*p*=0.02) (Figure 2).

We found a significant relationship between the healing percentage and time interval and treatment subgroups. Also, the healing rates in different groups were significant in different time intervals (*p*<0.001). According to the results, wound dimension mean in the treatment subgroups at different time intervals efficiently showed that the decrease rate of wound dimension in the *S. striata* subgroups was much more than that of the control and SSD subgroups (Figure 1). 

Our results indicated that the MIC for *S. striata* and SSD groups were respectively 45 mg/ml and 90 mg/ml, respectively while the MBC for these two were respectively 55 mg/ml and 100 mg/ml.

## DISCUSSION

Severe tissue damage caused by burns results in damage or loss of the tissue involved in some cases. Different organs have different healing powers.^24^As the skin is exposed to high temperature, the tissue collagen protein loses its spiral mode and turns into gelatin.^25^The SSD with anti-bacterial effects is widely used in burn centers. In treatment with SSD, the duration of therapy is long and undergoes tissue damages due to the prevention of keratinocyte and fibroblast proliferation and toxicity of these substances to cells.^6^

Use of conventional antibiotics has resulted into an increase in multi-drug resistant strains of *P. aeruginosa*, which is one of the most worrying opportunistic factors in nosocomial infections.^9^

Various studies were done to develop more effective dressings to improve the healing process and decrease the bacterial burden in burn wounds. Even medicinal plants were introduced in wound healing of burned injuries, traditional forms of medicine, especially herbal products, which were employed for centuries are under scientific investigation for their roles in wound healing.^10^^-^^14^

*S. striata *extract was shown to have antiseptic effects in treating infections caused by gram positive and negative bacteria and virus.^15^^,^^17^^19^ Our results indicate that unlike SSD, the effect of *S. striata e*xtract on *P. aeruginosa *infection in *in-vivo* conditions is much more than *in-vitro* conditions. We found a decrease in infection due to the effects of flavonol and flavonoid compounds of the *S. striata *extract in combination with cell wall structures or extracellular proteins.^19^ Our data indicate that epithelial formation was completed in the *S. striata* group before the 21_st _day, while irregular epithelialization was seen in the control group in the last time interval. 

These results can be due to the presence of iridoid glycoside compounds of *S. striata* that are capable of fibroblast growth and causing more collagen release and faster healing which is quite in contrast to the cytotoxic effects of SSD.^16^ In the current study, the degree of necrosis and inflammation in the *S. striata* group was very low. The anti-inflammatory effect can be due to the presence of phenyl propanoid glycosides seen in *S. striata* species which inhibited the activities of macrophages and production of chemical mediators and consequently reduced inflammation and subsequently promotes organization.^16^^,^^26^

The value of tissue repair in wounds treated with *S. striata* is much faster than other groups. In the *S. striata* group, the epidermis, collagen, hair follicles, and sebaceous gland were organized completely and systematically on the 14_th _day. Full recovery resulting from healing and anti-microbial properties of this extract led to a complete remission on the 14_th_ day in a way that the decrease in infection reduced the inflammation in the wound area. However, the presence of bacterial infection was a cause of inflammation increase, which resulted into tissue destruction and more inflammation in the wound area.^19^

Considering our findings, we can suggest that the anti-bacterial effects in synergy with the activating compounds of fibroblasts and keratinocytes were the most important factors in wound healing. The difference between anti-bacterial effects of *S. striata* and SSD in *in-vivo* and *in-vitro* showed the synergistic anti-bacterial and constructive effect of *S. striata* with respect to SSD. However, SSD had cytotoxic effects along with anti-microbial effects and its use led to an increased length of treatment.^24^ This study indicated that the ethanolic extract of *S. striata* with an anti-bacterial effect decreased the inflammation and necrosis in the burn wound areas and lead to collagen release and complete epithelialization in wound healing.

Our results indicated that ethanolic extract of *Scrophularia striata *could accelerate the wound healing and may be a good candidate for treating infected burn wounds because of strong anti-bacterial and healing effects.

## Note

Please cite this paper as:

Tanideh N, Haddadi MH, Rokni-Hosseini MH, Hossienzadeh M, Mehrabani D, Sayehmiri K, Koohi-Hossienabadi O. The Healing Effect of *Scrophularia Striata* on Experimental Burn Wounds Infected to *Pseudomonas Aeruginosa *in Rat. World J Plast Surg 2015;4(1):16-22.
